# Supplementary material for: Soft mode origin of charge ordering in superconducting kagome CsV3Sb5
Source: Nat Commun. 2026 May 30;17:4817. doi: 10.1038/s41467-026-73662-4 (PMC13222365; doi:10.1038/s41467-026-73662-4)
Supplement: Supplementary file 1 — Supplementary Information [file 41467_2026_73662_MOESM1_ESM.pdf]

Soft Mode Origin of Charge Ordering in Superconducting Kagome CsV_3Sb_5 - Supplementary Information

Philippa Helen McGuinness[1†], Fabian Henssler[1†], Manex Alkorta[2,3], Mark Joachim Graf von Westarp[1,4], Artem Korshunov[5], Alexei Bosak[5], Daisuke Ishikawa[6,7], Alfred Q. R. Baron[6,7], Michael Merz[1,8], Amir-Abbas Haghighirad[1], Maia G. Vergniory[9,10,11], Sofia-Michaela Souliou[1], Rolf Heid[1], Ion Errea[2,3,9], Matthieu Le Tacon[1*]

[1]Institute for Quantum Materials and Technologies, Karlsruhe Institute of Technology, Karlsruhe, 76021, Germany.
[2]Centro de Física de Materiales (CFM-MPC), CSIC-UPV/EHU, Donostia, 20018, Spain.
[3]Department of Applied Physics, University of the Basque Country (UPV/EHU), Donostia, 20018, Spain.
[4]Max Planck Institute for Solid State Research, Heisenbergstraße 1, D-70569 Stuttgart, Germany.
[5]ESRF, The European Synchrotron, 71, avenue des Martyrs, CS 40220 F-38043 Grenoble Cedex 9.
[6]Materials Dynamics Laboratory, RIKEN SPring-8 Center, Kouto 1-1-1, Sayo Hyogo 679–5148, Japan.
[7]Precision Spectroscopy Division, SPring-8/JASRI, Kouto 1-1-1, Sayo, Hyogo 679–5198, Japan.
[8]Karlsruhe Nano Micro Facility (KNMFi), Karlsruhe Institute of Technology, Kaiserstr. 12, 76131 Karlsruhe, Germany.
[9]Donostia International Physics Center (DIPC), Donostia, 20018, Spain.
[10]Département de Physique et Institut Quantique, Université de Sherbrooke, Sherbrooke, J1K 2R1 Québec, Canada.
[11]Regroupement Québécois sur les Matériaux de Pointe (RQMP), Québec H3T 3J7, Canada.

*Corresponding author(s). E-mail(s): matthieu.letacon@kit.edu;
†These authors contributed equally to this work.

Supplementary Note 1: Computational Approach

All the calculations presented throughout this manuscript are based on first principles. The Born-Oppenheimer energy (BOE) is studied at a density-functional theory (DFT) level using the Vienna Ab Initio Simulation Package (VASP) [1]. The reciprocal space is sampled by a Γ-centered 16×16×8 regular **k**-grid using a Methfessel-Paxton smearing of 0.1 eV, and a plane-wave energy cutoff of 400 eV. Projector augmented-wave (PAW) pseudopotentials were employed, with valence configurations $5s^2 5p^6 6s^1$, $3p^6 3d^4 4s^1$, and $5s^2 5p^3$ for Cs, V and Sb respectively. The out of plane Van der Waals character of the material is well described by the optB88-VdW [2] exchange-correlation functional.

The temperature-dependent dynamics of the system were calculated based on the stochastic self-consistent harmonic approximation (SSCHA) [3, 4]. Within this approximation, the frozen phonons are not limited to second derivatives of the BOE (the so-called harmonic phonons), but include anharmonicity and ionic fluctuations by variationally minimizing the temperature-dependent free energy of the system. To this end, the ionic distribution density of each normal mode is approximated by a Gaussian, characterized by its centroid position $\boldsymbol{\mathcal{R}}$ and force-constants $\boldsymbol{\Phi}$ as variational parameters. The ground-state ionic distribution obtained at each temperature is later used in the calculation of the anharmonic spectral function.

The phonon-phonon interaction is treated within the dynamical SSCHA theory [4, 5]. To achieve convergence of the phonon self-energies, the auxiliary force-constants $\boldsymbol{\Phi}(\mathbf{q})$ need to be computed in a $4\times4\times2$ **q**-grid. This made a purely ab-initio approach infeasible. Instead, a Gaussian approximation potential (GAP) [6] was used to calculate energies, forces, and stresses. The dataset, composed of 5000 configurations, was generated from physical SSCHA ionic distribution functions with a $2 \times 2 \times 2$ modulation of the high-symmetry phase. The GAP was self-consistently trained, with over 1000 configurations selected from the dataset for the final potential. The resulting highly accurate potential deviates from the dataset with a root mean square error of 0.13 meV/atom, 21.5 meV/Å, and 0.18 meV/Å^3 for energies, forces, and stresses.

The approximations made for the estimation of phonon linewidths and renormalization are explicitly discussed in the next section. The phonon-phonon interaction is calculated within the Bubble approximation, using a $24 \times 24 \times 24$ **k**-grid and a $\eta = 5\,\mathrm{cm}^{-1}$ smearing. The electron-phonon linewidth is obtained from density functional perturbation theory (DFPT) implemented in the Quantum Espresso package [7, 8]. A $16 \times 16 \times 8$ **k**-grid was used for reciprocal space sampling, with a Methfessel-Paxton smearing of 0.01 Ry. The deformation potential was computed using a $2 \times 2 \times 2$ **q**-grid with an electron-phonon smearing of 0.01 Ry. The ultra-soft pseudopotentials employed were generated by Dal Corso [9], with valence configurations of $5s^2 5p^6 6s^1$, $3s^2 3p^6 3d^3 4s^2$, and $5s^2 5p^3$ for Cs, V and Sb respectively. The

optB88-vdW [2] functional was used for exchange and correlation, previously shown to be accurate for this material.

Supplementary Note 2: Anharmonic spectral functions combined with electron-phonon interaction

To accurately describe the IXS measurements, the dynamic structure factor of each vibrational mode must be computed for the wave vector of interest. The structure factor estimates how the spectral weight of a mode is renormalized in reciprocal space. At Γ, where renormalization vanishes, the spectral weight is closely related to the density of states. This is not the case away from the zone center, where the renormalization of each mode's spectral weight depends on its polarization vector and the wave vector $\mathbf{Q} = \mathbf{q} + \mathbf{G}$ at which the phonon is, where $\mathbf{G}$ is a reciprocal lattice vector. From literature [10, 11], the one-phonon dynamic structure factor is expressed as

$$F_\mu(\mathbf{Q}) = \sum_{a,\alpha} \frac{f_a(\mathbf{Q})e^{-W_a}}{\sqrt{2M_a\omega_\mu(\mathbf{q})}}\, Q_\alpha \cdot \epsilon^\alpha_{\mu,a}(\mathbf{q})\, e^{iQ_\alpha \cdot R^\alpha_a} \tag{1}$$

Here, M_a is the mass of the atom with index a,α labels the cartesian index, and $\omega_\mu(\mathbf{q})$ is the frequency of mode μ. $f_a(\mathbf{Q})$ is the atomic form factor, given by the Fourier transform of the atomic electron density. This describes how a single atom scatters X-rays as a function of momentum transfer $\mathbf{Q}$. $e^{-W_a(\mathbf{Q})}$ is the Debye–Waller factor, which quantifies the reduction in Bragg peak intensity due to thermal vibrations. The Debye–Waller exponent is $W_a(\mathbf{Q}) = \frac{1}{2}\langle(\mathbf{Q} \cdot \mathbf{u}_a(\mathbf{q}))\rangle$, with $\mathbf{u}_a(\mathbf{q})$ the atomic displacement vector. $\boldsymbol{\epsilon}_\mu(\mathbf{q})$ is the polarization vector of mode μ, and $\mathbf{R}_a$ is the atomic position.

To rewrite the above expression solely in terms of the phonon spectra, we apply two simplifying approximations. First, we assume that the atomic form factor is dispersionless and identical for all atoms. Second, we assume that ionic displacements are small enough not to affect the dynamic structure factor, i.e. $\langle \mathbf{Q} \cdot \mathbf{u}_a(\mathbf{q})\rangle \ll 1$. With these approximations, the dynamic structure factor simplifies to

$$F_\mu(\mathbf{Q}) = \sum_{a,\alpha} \frac{1}{\sqrt{2M_a\omega_\mu(\mathbf{q})}}\, Q_\alpha \cdot \epsilon^\alpha_{\mu,a}(\mathbf{q})\, e^{iQ_\alpha \cdot R^\alpha_a}\,, \tag{2}$$

which is the final expression for the dynamic structure factor employed in this manuscript.

In addition, vibrational modes cannot be treated as quasiparticles with infinite lifetime. Here, we approximate the temperature-dependent phonon spectral function as a sum of independent modes with Lorentzian distribution, where the linewidth is determined by both phonon–phonon and electron–phonon scattering.

Thus, the phonon spectral function is written as

$$
\begin{aligned}
A(\omega, \mathbf{Q}, T) &= \sum_{\mu} |F_\mu(\mathbf{Q})|^2 A_\mu(\omega, \mathbf{q}, T) \\
A_\mu(\omega, \mathbf{q}, T) &= \frac{1}{\pi} \frac{\Gamma_\mu(\mathbf{q}, T)}{[\omega - \Omega_\mu^{ph-ph}(\mathbf{q}, T)]^2 + [\Gamma_\mu(\mathbf{q}, T)]^2} \,,
\end{aligned}
\tag{3}
$$

where $\Omega_\mu^{ph-ph}(\mathbf{q}, T)$ is the Lorentzian peak frequency of mode μ, and

$$
\Gamma_\mu(\mathbf{q}, T) = \Gamma_\mu^{ph-ph}(\mathbf{q}, T) + \Gamma_\mu^{el-ph}(\mathbf{q}, T) \,, \tag{4}
$$

is the half width at half maximum (HWHM). Note that the the dynamic structure factor $F_\mu(\mathbf{Q})$ is calculated for the anharmonicity corrected phonon frequencies Ω_μ^{ph-ph}. All approximations used to estimate the temperature dependence of phonon–phonon and electron–phonon linewidths are explained in detail in the following subsections. To follow the notation in the literature, the temperature dependence of the phonon spectra will be left implicit and reintroduced in the final expression of the linewidths.

Phonon self-interaction within the bubble approximation

The phonon-phonon interaction is calculated within the dynamical formulation of the SSCHA theory [5]. In the current implementation, the phonon-phonon self-energy is approximated within the bubble approximation into

$$
\mathbf{\Pi}^{ph-ph}(\mathbf{q}, \omega) \approx \mathbf{\Pi}^{(B)}(\mathbf{q}, \omega) = \mathbf{\Phi}^{(3)}(\mathbf{q}) : \mathbf{\Lambda}(\omega) : \mathbf{\Phi}^{(3)}(\mathbf{q}) \,, \tag{5}
$$

where $\mathbf{\Phi}^{(3)}(\mathbf{q})$ are the third order force constants, and $\mathbf{\Lambda}(\omega)$ is a fourth order tensor dependent on the SSCHA auxiliary frequencies and polarization vectors. For simplicity we used the compacted formalism, where $\mathbf{X} : \mathbf{Y}$ stands for the double summation in the last two indices of $\mathbf{X}$ and the first two indices of $\mathbf{Y}$.

In order to overcome convergence issues in the calculation of $\mathbf{\Pi}^{(B)}(\mathbf{q}, \omega)$ a small but finite smearing η is employed along with a interpolated fine phonon $\mathbf{k}$-grid. This is mathematically expressed as

$$
\begin{aligned}
\mathbf{\Pi}^{(B)}(\mathbf{q}, \omega + i\eta) &= \sum_{\mathbf{k}_1\mathbf{k}_2} \sum_{\mathbf{G}} \delta_{\mathbf{G}, \mathbf{q}+\mathbf{k}_1+\mathbf{k}_2} \cdot \\
\cdot \mathbf{\Phi}^{(3)}(-\mathbf{q}, -\mathbf{k}_1, -\mathbf{k}_2) &: \mathbf{\Lambda}(\omega + i\eta, -\mathbf{k}_1, -\mathbf{k}_2, \mathbf{k}_1, \mathbf{k}_2) : \mathbf{\Phi}^{(3)}(\mathbf{q}, \mathbf{k}_1, \mathbf{k}_2) \,,
\end{aligned}
\tag{6}
$$

where $\mathbf{k}_i$ are phonon wave vectors, and $\mathbf{G}$ is a reciprocal lattice vector.

In the general case, the self-energy $\mathbf{\Pi}^{(B)}(\mathbf{q}, \omega)$ is a Hermitian matrix with components $\Pi_{\mu\nu}^{(B)}(\mathbf{q}, \omega)$ in the phonon mode basis, where μ and ν label the vibrational branches at wave vector $\mathbf{q}$. Therefore, in the presence of anharmonicity, hybridization occurs, and vibrational modes are no longer well defined quasiparticles. In order to combine the SSCHA dynamical theory with density functional perturbation theory

electron-phonon calculations, we need to keep track of the contribution to the spectra of each vibrational mode. This is achieved under the no mode-mixing approximation. By neglecting the off-diagonal elements $\Pi^{(B)}_{\mu\nu}(\mathbf{q},\omega)$ with $\mu \neq \nu$, and assuming that each phonon mode propagates independently, the Green function then becomes diagonal in the mode index μ. Consequently, the mode-resolved spectral function simplifies to

$$A^{ph-ph}_{\mu}(\mathbf{q},\omega) = -\frac{1}{\pi}\,\mathrm{Im}\left[\frac{1}{(\omega+i\eta)^2-\omega^2_{\mu}(\mathbf{q})-\Pi^{(B)}_{\mu\mu}(\mathbf{q},\omega)}\right], \tag{7}$$

which can be expressed with good approximation as a Lorentzian function

$$A^{ph-ph}_{\mu}(\mathbf{q},\omega) \approx \frac{1}{\pi}\frac{\Gamma^{ph-ph}_{\mu}(\mathbf{q})}{[\omega-\Omega^{ph-ph}_{\mu}(\mathbf{q})]^2+[\Gamma^{ph-ph}_{\mu}(\mathbf{q})]^2}, \tag{8}$$

centered at the frequency

$$\Omega^{ph-ph}_{\mu}(\mathbf{q}) = \mathrm{Re}\left\{\sqrt{\omega^2_{\mu}(\mathbf{q})+\Pi^{(B)}_{\mu\mu}(\mathbf{q},\omega)}\right\}, \tag{9}$$

with a half width at half maximum (HWHM) of

$$\Gamma^{ph-ph}_{\mu}(\mathbf{q}) = -\mathrm{Im}\left\{\sqrt{\omega^2_{\mu}(\mathbf{q})+\Pi^{(B)}_{\mu\mu}(\mathbf{q},\omega)}\right\}. \tag{10}$$

Even though we followed the original notation of the dynamical SSCHA theory, the auxiliary phonon spectra $\omega_{\mu}(\mathbf{q})$ and $\Pi_{\mu\mu}(\mathbf{q},\omega)$ are temperature dependent. Thus it is more convenient to rewrite the phonon linewidth obtaining the first term of Eq. 4 as

$$\Gamma^{ph-ph}_{\mu}(\mathbf{q},T) = -\mathrm{Im}\left\{\sqrt{\omega^2_{\mu}(\mathbf{q},T)+\Pi^{(B)}_{\mu\mu}(\mathbf{q},\omega,T)}\right\}. \tag{11}$$

Linear response electron-phonon interaction

The electron-phonon interaction is computed using density functional perturbation theory implemented in the Quantum Espresso package. In linear response, the electron-phonon self-energy is given by

$$\Pi^{el-ph}_{\mu}(\mathbf{q}) = \frac{1}{N}\sum_{n,m}\sum_{k}|g^{\mu}_{n,k}(\mathbf{k},\mathbf{k}+\mathbf{q})|^2\frac{f_{n\mathbf{k}}-f_{m\mathbf{k}+\mathbf{q}}}{\varepsilon_{n\mathbf{k}}-\varepsilon_{m\mathbf{k}+\mathbf{q}}+\omega+i\delta}, \tag{12}$$

where n and m label electronic states, $f_{n\mathbf{k}}$ corresponds to the occupation of electronic state n with wave-number $\mathbf{k}$, and $g^{\mu}_{n,m}(\mathbf{k},\mathbf{k}+\mathbf{q})$ are the electron-phonon matrix elements defined as

$$g^{\mu}_{n,m}(\mathbf{k},\mathbf{k}+\mathbf{q}) = \sum_{\alpha,i}\frac{1}{\sqrt{2M_i\omega_{\lambda}(\mathbf{q})}}\epsilon^{\alpha}_{\lambda,i}\langle n\mathbf{k}|\left[\frac{\partial V_{KS}}{\partial u^{\alpha}_{i}(\mathbf{q})}\right]_0|m\mathbf{k}+\mathbf{q}\rangle\,. \tag{13}$$

The derivatives of the Kohn-Sham potential V_{KS} respect to ionic displacements $u_a^\alpha(\mathbf{q})$ will be relevant in the later. Evaluating the self-energy at the bare frequencies ω_μ, and truncating to first order, the half width at half maximum (HWHM) of the phonons due to scattering with electrons is given by

$$\Gamma^{el-ph}(\mathbf{q}) = -\mathrm{Im}[\Pi_\mu^{el-ph}(\mathbf{q}, \omega_\mu(\mathbf{q}))] \,. \tag{14}$$

In the $T = 0\,\mathrm{K}$ limit for electronic temperature, and assuming that phonon energies are much smaller than electron energies, where it can be shown that

$$\Gamma_\mu^{el-ph}(\mathbf{q}) = \frac{2\pi\omega_\mu(\mathbf{q})}{N} \sum_{n,m} \sum_{k} |g_{n,k}^{\mu}(\mathbf{k}, \mathbf{k}+\mathbf{q})|^2 \delta(\varepsilon_{n\mathbf{k}} - \varepsilon_F)\delta(\varepsilon_{m\mathbf{k}+\mathbf{q}} - \varepsilon_F) \,, \tag{15}$$

which can be rewritten into

$$\Gamma_\mu^{el-ph}(\mathbf{q}, T) = \frac{\pi}{N} \sum_{ab} \sum_{\alpha,\beta} \frac{1}{\sqrt{M_a M_b}} \epsilon_{\mu,a}^{\alpha}(\mathbf{q}, T) \Delta_{ab}^{\alpha\beta}(\mathbf{q}) \epsilon_{\mu,a}^{\alpha}(-\mathbf{q}, T) \,, \tag{16}$$

where $\Delta_{ab}^{\alpha\beta}(\mathbf{q})$ is given by

$$\Delta_{ab}^{\alpha\beta}(\mathbf{q}) = \sum_{k} \sum_{nm} \langle n\mathbf{k}| \frac{\partial V_{KS}}{\partial u_a^\alpha(\mathbf{q})} |m\mathbf{k}+\mathbf{q}\rangle \langle m\mathbf{k}+\mathbf{q}| \frac{\partial V_{KS}}{\partial u_b^\beta(\mathbf{q})} |n\mathbf{k}\rangle \; \delta(\varepsilon_{n\mathbf{k}} - \varepsilon_F)\delta(\varepsilon_{m\mathbf{k}+\mathbf{q}} - \varepsilon_F) \,. \tag{17}$$

This definition of electron-phonon linewidth allows to combine density functional perturbation theory based electron-phonon calculations with an arbitrary phonon spectra. This is extremely useful as it allows to resolve the temperature evolution of the linewidth of a certain mode, as all temperatures can be resolved from a unique calculation of $\Delta_{ab}^{\alpha\beta}(\mathbf{q})$.

Supplementary Note 3: Predicting the observability of the phonon collapse

Even though a recent study predicted that the CDW phase transition is a consequence of a phonon collapse at the L wave vector [12], due to large electron-phonon coupling, its observability remains an open question. To answer this question, we calculated the anharmonic phonon spectral functions of CsV_3Sb_5 as a function of temperature (see Supplementary Note 2). These calculations were performed by using Eq. 3, which includes anharmonicity, phonon-phonon interactions and linear response electron-phonon coupling as well as the phonon resolved dynamic structure factor. This calculations suggest that, even in the presence of high anharmonicity, at the $\mathbf{G} = (1, 0, 3)$ Brillouin zone, the phonon softening at the ML direction is observable.

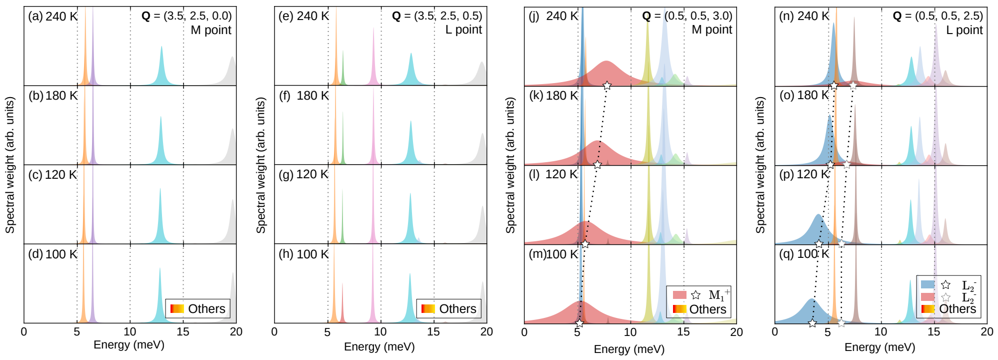

Supplementary Figure 1 (a-d) Anharmonic spectral function at the wave vector $\mathbf{Q} = (3.5, 2.5, 0)$ computed at 240 K (a), 180 K (b), 120 K (c) and 100 K (d). (e-h), (j-m) and (n-q) same as (a-d) for the wave vectors $\mathbf{Q} = (3.5, 2.5, 0.5)$, $\mathbf{Q} = (0.5, 0.5, 3)$ and $\mathbf{Q} = (0.5, 0.5, 2.5)$ respectively. When a phonon renormalization is observable the energies are highlighted.

Supplementary Note 4: Avoided crossing of $\mathbf{L}_2^-$ modes

The stability and dynamics of CsV_3Sb_5 are well understood within the SSCHA framework, where a L_2^- phonon becomes dynamically unstable not exactly at T_{CDW}, but at lower temperatures [13]. However, in apparent discrepancy with experiments, theory predicts two L_2^- modes softening while only one can be fitted with the experimental resolution. We attribute this phenomenon to the strong hybridization between these soft modes.

Both modes belong to the L_2^- irreducible representation, forbidding by symmetry a crossing of the phonon bands. However, the hybridization between these two bands is not forbidden. A clear way to look at this hybridization is the dynamic structure factor $F_\mu(\mathbf{q})$. The dynamic structure factor quantifies how the intensity of a vibrational mode varies in reciprocal space, which is extremely useful for filtering non-relevant modes, whose structure factor remains invariant in the absence of hybridization. Hybridization between modes is likely to occur close to a phase transition. When the free energy is close to developing a new ground state, the shape of the BOE landscape changes in the direction of the developing local minima. Supplementary Fig. 2 shows that while the structure factor of the rest of the modes is perfectly constant towards the phase transition, the two L_2^- modes show strong renormalization. The low energy mode gains spectral weight in the cooling, while the high energy mode disappears. This phenomenon leads to a constant evolution of the total spectral weight with temperature (see Supplementary Fig. 2).

As is clear from Figs. 3 and 4 in the main text, the low-energy spectra of CsV_3Sb_5 in the studied Brillouin zone contains multiple overlapping phonons, which makes fitting challenging. In particular, there is an intense phonon at 5.3 meV, which is very similar to the energy of the avoided crossing. This, combined with the experimental resolution and the dramatic changes in structure factor described in Supplementary Fig. 2-b, obscures the avoided crossing behavior and makes it impossible to reliably distinguish this behavior from a single phonon softening from high energy when fitting. This not

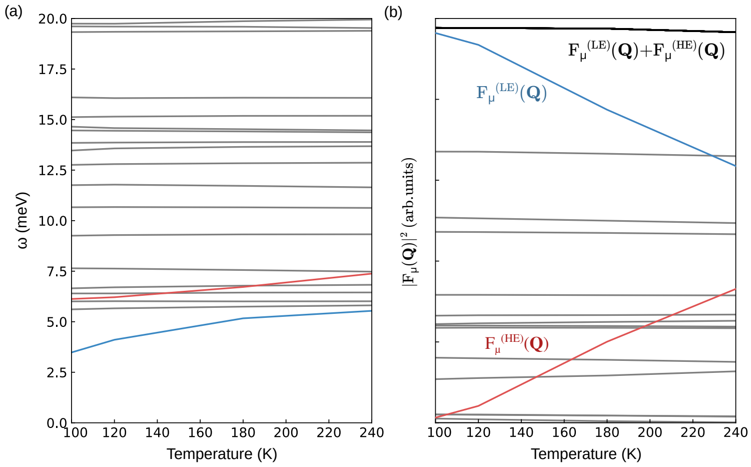

Supplementary Figure 2 Avoided crossing and hybridization of the L_2^- modes. (a) Temperature evolution of the phonon frequencies $\omega_\mu(\mathbf{q})$, highlighting the L_2^- high energy (HE) and low energy (LE) modes in color. (b) Dynamic structure factor of $F_\mu(\mathbf{Q})$ calculated at $\mathbf{Q} = (0.5, 0.5, 2.5)$. The dynamic structure factor of the L_2^- modes is highlighted, as well as their sum.

only resolves the discrepancy between theory and experiment, but it even reinforces the existence of soft phonon physics in CsV_3Sb_5.

Supplementary Note 5: Dynamical structure factor calculations

In an IXS experiment, the measured intensity is determined by the product of the phonon spectral function and the one-phonon dynamical structure factor. The spectral function contains the intrinsic phonon properties, such as energy, damping, and temperature-dependent renormalization, whereas the dynamical structure factor acts as a matrix element that governs how strongly a given phonon mode contributes to the scattered intensity at a specific momentum transfer. For a phonon branch ν with wavevector $\mathbf{q}$, the one-phonon dynamical structure factor can be written as

$$S(\mathbf{Q},\omega) \propto \sum_\nu \frac{1}{\omega_{\mathbf{q}\nu}} \left| \sum_d \frac{f_d(\mathbf{Q})}{\sqrt{M_d}} e^{-W_d(\mathbf{Q})} (\mathbf{Q}\cdot\mathbf{e}_{d,\mathbf{q}\nu}) e^{i\mathbf{Q}\cdot\mathbf{r}_d} \right|^2 \delta(\omega - \omega_{\mathbf{q}\nu}), \tag{18}$$

where $f_d(\mathbf{Q})$ is the atomic form factor of atom d, M_d its mass, W_d the Debye–Waller factor, $\mathbf{e}_{d,\mathbf{q}\nu}$ the phonon eigenvector, and $\mathbf{r}_d$ the atomic position. Because the intensity depends on the projection $\mathbf{Q}\cdot\mathbf{e}_{d,\mathbf{q}\nu}$ and interference between atomic contributions, the structure factor can vary strongly between symmetry-equivalent points in different Brillouin zones.

Using phonon eigenvectors obtained from first-principles calculations, we evaluated the dynamical structure factor across multiple experimentally accessible Brillouin zones. The result of this analysis for the M and L points is shown in Supplementary

Fig. 3 and revealed that the unstable phonon branch carries weak intensity most of the Brillouin zones, encompassing those investigated in previous work [14, 15]. In a few Brillouin zones, the structure factor is strongly enhanced. This is the case near the M and L points around the Γ_{103} zone which have been chosen for the present IXS measurements.

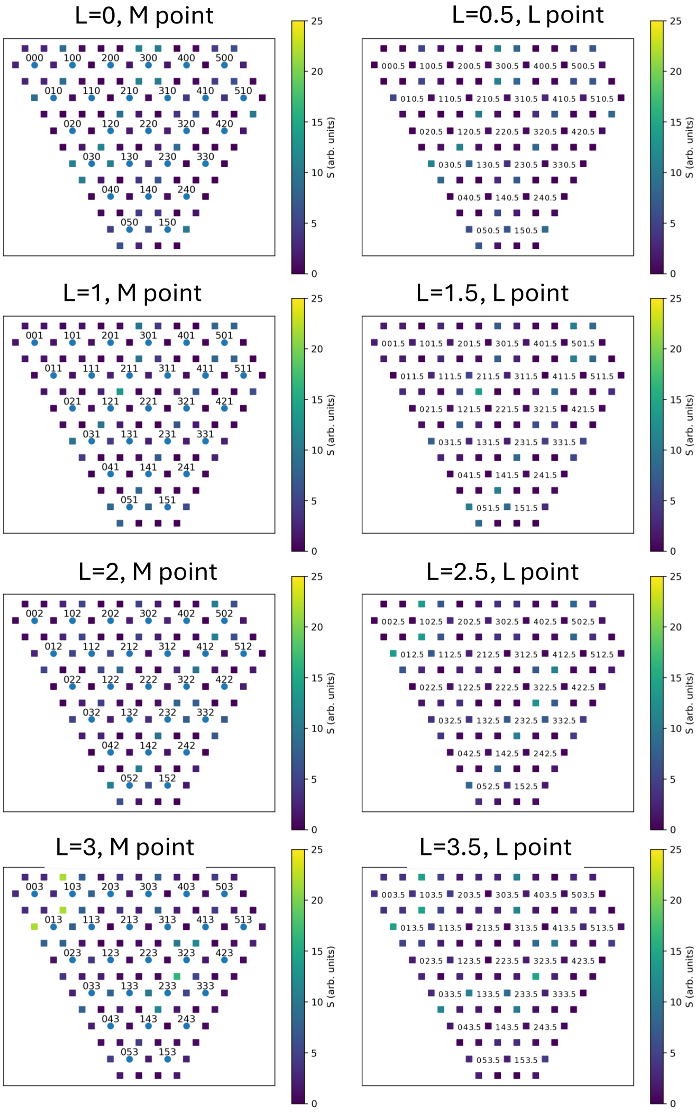

Supplementary Figure 3 Calculated structure factor for the unstable phonon branch at representative M points in the (H,K,0), (H,K,1), (H,K,2), and (H,K,3) reciprocal planes, and at L points in the (H,K,0.5), (H,K,1.5), (H,K,2.5), and (H,K,3.5) planes.

Supplementary Note 6: Temperature dependence of elastic intensity across T_{CDW}

When the intensity at zero energy is measured across the CDW transition, as displayed in Supplementary Figure 4, there is a small hysteresis of around 0.5 to 1 K between the transition temperatures on cooling and heating for both the M and L points, supporting the conclusion from thermodynamic studies about the first order nature of the transition, as discussed in the main text.

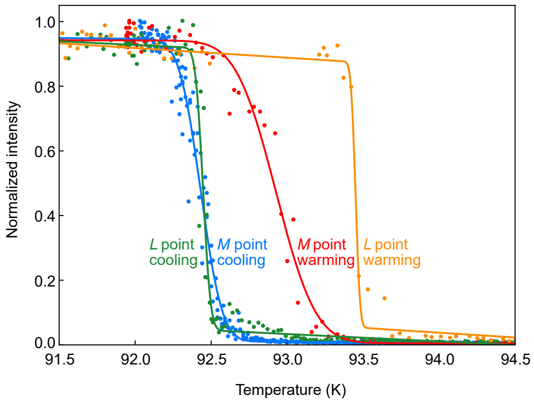

Supplementary Figure 4 Temperature dependence of the elastic intensity across the CDW transition. Normalized intensity of the zero-energy (elastic) peak measured at M ($\mathbf{Q} = (0.5,\ 0.5,\ 3)$) and L ($\mathbf{Q} = (0.5,\ 0.5,\ 3.5)$) around the CDW transition temperature, on both cooling and warming. The lines are fits to the data using an error function with a quadratic background.

Supplementary Note 7: Temperature dependence of phonon energies at $\mathbf{Q} = (0.75,\ 0.25,\ 3)$

As discussed in the main text, when moving away from the M point towards Γ, the phonon softening weakens. Supplementary Figure 5 displays the fitted phonon energies at the midpoint along Γ-M, at $\mathbf{Q} = (0.75,\ 0.25,\ 3)$. There is no clear temperature dependence of the phonon energy for any of the fitted phonon branches, and, in particular, no evidence of phonon softening, setting an upper limit on the scale of the softening effect in-plane.

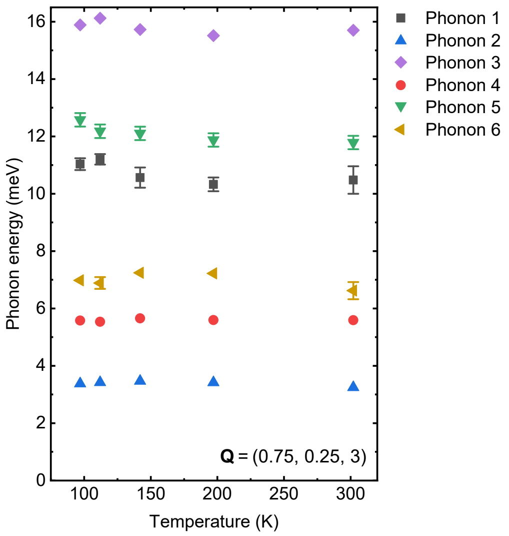

Supplementary Figure 5 Temperature dependence of the energies of the six fitted phonons at $\mathbf{Q} = (0.75,\ 0.25,\ 3)$ the midpoint of Γ-M, between 97 K and 300 K.

Supplementary Note 8: Simulation of the effect of temperature at the M and L points

From the spectra measured at room temperature, it is straightforward to simulate the temperature dependence expected if the spectra were governed solely by the Bose factor, and to compare this with the actual data.

The dynamic structure factor $S(\mathbf{Q}, \omega, T)$ is related to the imaginary part of the dynamic susceptibility $\chi''(\mathbf{Q}, \omega, T)$ through

$$S(\mathbf{Q}, \omega, T) = \frac{n(\omega, T) + 1}{\pi} \chi''(\mathbf{Q}, \omega, T),$$

where $n(\omega, T) = 1/(\exp(\hbar\omega/k_B T) - 1)$ is the Bose factor [11].

We therefore extract $\chi''(\mathbf{Q}, \omega, 302\,\mathrm{K})$ by subtracting a resolution-limited elastic line and correcting the resulting spectrum by the Bose factor at 302 K (the subtraction of the elastic line plays only a minor role in this procedure). Assuming that $\chi''(\mathbf{Q}, \omega)$ itself does not change with temperature, spectra at any other temperature can then be simulated by applying the corresponding Bose factor.

A direct comparison between the simulated temperature evolution and the experimental data is shown in Supplementary Fig. 6 for the M and L points. The comparison reveals a clear redistribution of spectral weight in the measured spectra that goes well beyond the trivial Bose-factor enhancement of the elastic line.

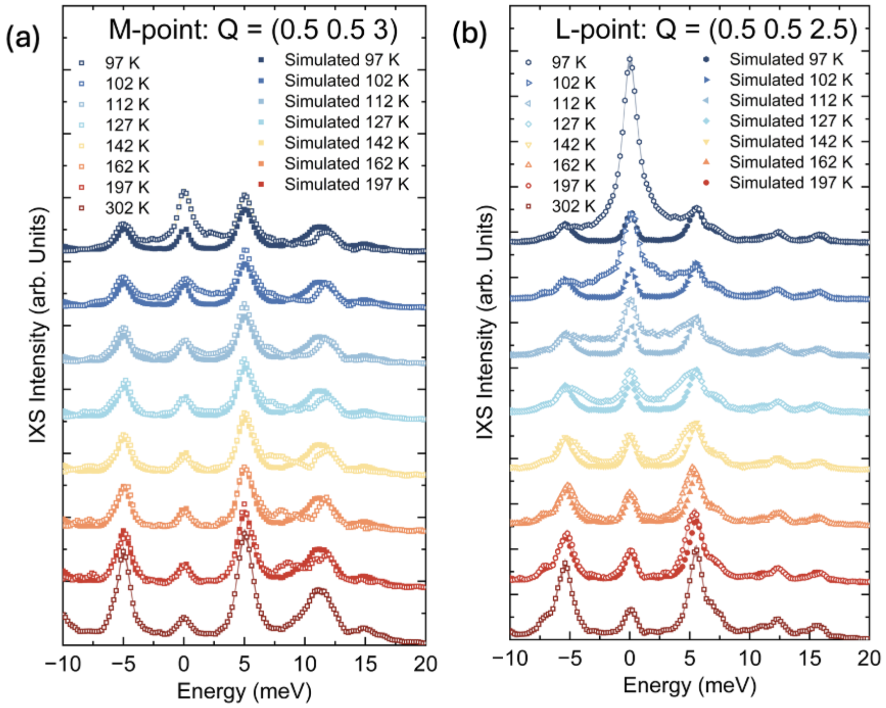

Supplementary Figure 6 Comparison between experimental spectra measured at the M and L points at various temperatures and spectra simulated assuming only a trivial Bose-factor temperature dependence inferred from the room-temperature data. The deviations clearly indicate a redistribution of spectral weight beyond the Bose-factor effect.

Supplementary Note 9: Additional fitting details

The phonons were fitted using a damped harmonic oscillator function, $S_j(Q,E)$ which is a function of the wave vector Q and the energy E, weighted by the Bose factor. For the jth phonon,

$$S_j(Q,E) = \frac{A_j}{\pi} \frac{1}{1-e^{-\frac{E}{k_\mathrm{B}T}}} \frac{4\Gamma_j E}{\left(E^2-(E_j^2+\Gamma_j^2)\right)^2+4(\Gamma_j E)^2}, \tag{19}$$

where T is the temperature, A_j is the phonon structure factor, E_j the damped phonon energy of the jth phonon and Γ_j is the damping rate (the HWHM of the phonon). The undamped phonon energy (described simply as the phonon energy in the main text) is then $E_{0j} = \sqrt{E_j^2+\Gamma_j^2}$. The width Γ_B of the unstable phonon branch, as is clear from the spectra displayed in Figs. 3 and 4 in the main text, is strongly temperature dependent, particularly at the M point. Supplementary Fig. 7 displays the behavior of the fitted energy E_B and width Γ_B as a function of temperature for the M, U and L points. The error bars σ_E in these plots correspond to the fit σ_Γ uncertainties.

With decreasing temperature, there is a clear increase in the width at the M and U points. The width at the L point is more uncertain at lower temperatures due to the overlap with the elastic peak, but also shows a weaker increase with decreasing temperature.

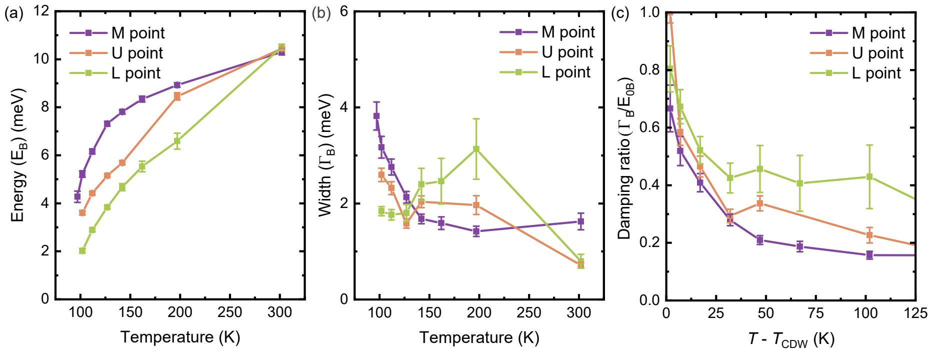

Supplementary Figure 7 Temperature dependence of the fitted energy (a), width (b, ≈HWHM) and damping ratio (c, Γ/E_0) of the softening phonon (Phonon B in the main text) at the M ($\mathbf{Q}$ = (0.5, 0.5, 3)), U ($\mathbf{Q}$ = (0.5, 0.5, 2.75)) and L ($\mathbf{Q}$ = (0.5, 0.5, 2.5)) points.

We note that the width at the L point at 162 K gets out of the trend and shows larger uncertainty. This is because the maximum intensity of the soft mode lies close to that of a strong phonon near 5 meV. In the intermediate temperature range (140-180 K), the spectral weight of the anomalous branch is distributed across two modes. As shown by the calculations (Supplementary Figure . 2), these two L_2^- modes undergo an avoided crossing and exchange spectral weight. Because both branches carry significant structure factor, their spectral overlap leads to an apparently larger effective linewidth when modeled with independent damped harmonic oscillators. The increased fitted width in this range is therefore qualitatively consistent with the theoretical picture of hybridization and spectral weight redistribution.

At lower temperatures (below ~120 K), the situation changes qualitatively: the upper branch loses most of its structure factor while the lower branch continues to soften. Once the soft branch dominates the spectral weight, the fitting becomes more stable as spectral overlap is reduced. In this regime the linewidth extracted from the DHO model should not be interpreted as a direct measure of the phonon lifetime; rather, the mode approaches an overdamped regime. Close to T_{CDW}, the growing elastic line also prevents a reliable extraction of the phonon energy.

Supplementary Note 10: Intensity of the CDW precursor along *L*-*M*

The intensity of the precursor of the CDW in the thermal diffuse scattering (TDS) measurements varies as a function of the out of plane momentum, as discussed in the main text. Supplementary Fig. 8 shows in-plane reconstructions of the TDS at 95 K

centered at points along the line $\mathbf{Q} = (0,\ 0,\ L)$. The precursor is sharpest and most intense at the L point, $\mathbf{Q} = (0.5,\ 0.5,\ 2.5)$ and equivalents, and weakens continuously along the line L-M, becoming only weakly visible at the M point (Supplementary Fig. 8). This is in agreement with the softening of the unstable phonon mode (Fig. 5b in the main text) and the growth of the elastic line (Fig. 5c in the main text), both of which are strongest at the L point, and therefore reinforces that the L point is the leading instability.

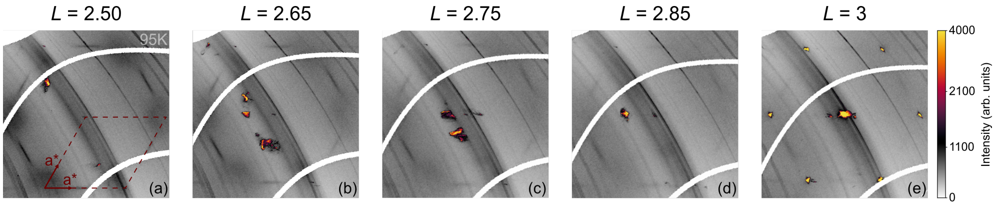

Supplementary Figure 8 In-plane diffuse scattering maps measured at 95 K at points centered along the line $\mathbf{Q} = (0,\ 0,\ L)$.

Supplementary Note 11: Transfer of spectral weight measured with lower resolution IXS

Supplementary Figure 9 is based on a series of IXS spectra recorded on ESRF beamline ID28 along the reciprocal-space path connecting the Γ, M, L, A points, with an energy resolution of 3 meV. The spectra were interpolated to visualize the momentum dependence of the IXS intensity, which is directly proportional to the dynamic structure factor $S(\mathbf{Q}, \omega, T)$, at 300 K (Supplementary Fig. 9-a) and 94 K (Supplementary Fig.9-c).

The dynamic structure factor $S(\mathbf{Q}, \omega, T)$ is related to the imaginary part of the dynamic susceptibility $\chi''(\mathbf{Q}, \omega, T)$ through

$$S(\mathbf{Q}, \omega, T) = \frac{n(\omega, T)}{\pi} \chi''(\mathbf{Q}, \omega, T),$$

where $n(\omega, T) = 1/(\exp(\hbar\omega/k_B T) - 1)$ is the Bose factor [11]. Any temperature dependence in $S(\mathbf{Q}, \omega, T)$ may therefore arise either from the trivial Bose-factor scaling or from the intrinsic temperature dependence of $\chi''(\mathbf{Q}, \omega, T)$. To disentangle these contributions, we extracted $\chi''(\mathbf{Q}, \omega, 300\,\mathrm{K})$ by fitting $S(\mathbf{Q}, \omega, 300\,\mathrm{K})$ with a series of DHOs, subtracting the resolution-limited elastic component, and dividing by the 300 K Bose factor. This was then used to simulate $S(\mathbf{Q}, \omega, 94\,\mathrm{K})$ under the assumption that the temperature dependence arises solely from the Bose factor. Assuming a temperature-independent elastic line, the resulting simulated spectra are shown in Supplementary Fig. 9-b. Subtracting this simulation from the measured spectra at 94 K yields Fig. 9-d. Interestingly, around $10 - 12\,\mathrm{meV}$, a small loss of spectral weight can be seen (blue area in Supplementary Fig. 9-d), whereas in contrast, a clear enhancement of

the elastic line along the M-L direction - most pronounced at the L point - as well as a significant increase in intensity below 5 meV (red area in Fig. 9-d). This indicates a transfer of spectral weight from the high to the low-energy part of the phonon spectrum, reflecting genuine changes in the dynamical susceptibility. These have been resolved with the higher-resolution measurements done at SPring-8 presented in the main manuscript and which have shown that this spectral weight transfer corresponds to the softening of the branch connecting the M_1^+ and L_2^- modes.

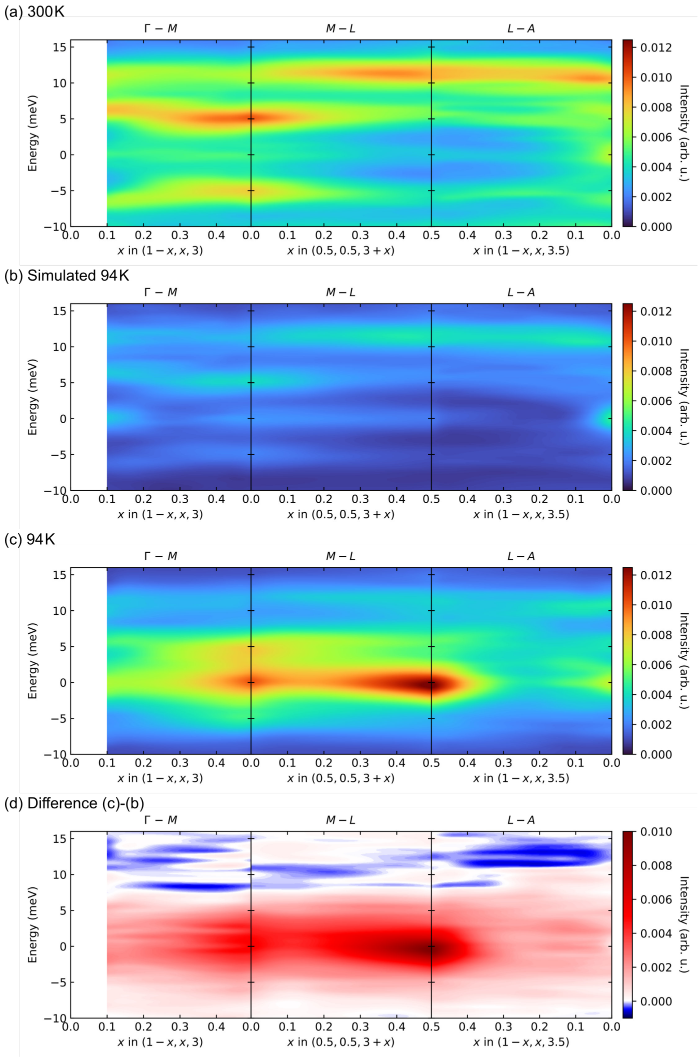

Supplementary Figure 9 Inelastic spectra taken with 3 meV resolution at beamline ID28 (ESRF). (a) 300 K raw data, (b) simulated 94 K data based on 300 K data, (c) 94 K raw data, (d) difference between (c) and (b).

Supplementary Note 12: Momentum dependence of electron-phonon coupling

Supplementary Figure 10 displays the momentum dependence of the linewidth due to the electron-phonon coupling along the M-L path. The anharmonic phonon dispersion obtained at 100 K from the peaks of the spectral functions is shown with thin purple lines. All modes in the color plot are depicted with the same artificial broadening, but the amplitude of the peak is proportional to the linewidth given by the electron-phonon coupling, thus, the color scheme signals the strength of this interaction. The figure clearly shows that the mode with the largest electron-phonon coupling at the M point is the softened M_1^+ and at the L point both L_2^- modes that show avoided crossing as discussed above. The continuation of these two modes in the M-L path also shows a large electron-phonon coupling. The agreement between the region of strong electron-phonon coupling and the region where phonon softening is observed provides further evidence that anisotropic EPC drives the CDW in CsV_3Sb_5.

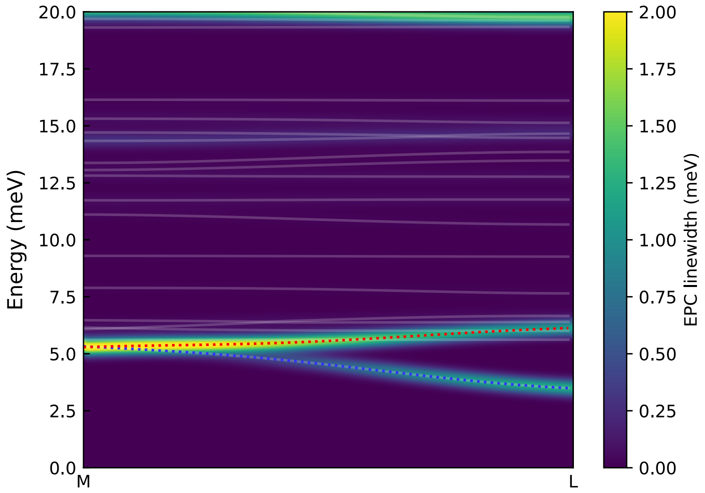

Supplementary Figure 10 Momentum dependence of the EPC along M-L. The anharmonic phonon dispersion obtained at 100 K with the SSCHA depicted with thin purple lines corresponds to the peaks of the spectral functions. The amplitude of the peaks in the color plot is proportional to the linewidth given by the electron-phonon coupling. The peaks of the spectral function of the L_2^- showing avoided crossing are highlighted with colored dashed lines.

Supplementary References

[1] Kresse, G. & Furthmüller, J. Efficient iterative schemes for ab initio total-energy calculations using a plane-wave basis set. *Physical Review B* **54**, 11169–11186 (1996).

[2] Klimeš, J., Bowler, D. R. & Michaelides, A. Chemical accuracy for the van der Waals density functional. *Journal of Physics: Condensed Matter* **22**, 022201 (2009).

[3] Errea, I., Calandra, M. & Mauri, F. Anharmonic free energies and phonon dispersions from the stochastic self-consistent harmonic approximation: Application to platinum and palladium hydrides. *Physical Review B* **89**, 064302 (2014).

[4] Monacelli, L. *et al.* The stochastic self-consistent harmonic approximation: Calculating vibrational properties of materials with full quantum and anharmonic effects. *Journal of Physics: Condensed Matter* **33**, 363001 (2021).

[5] Bianco, R., Errea, I., Paulatto, L., Calandra, M. & Mauri, F. Second-order structural phase transitions, free energy curvature, and temperature-dependent anharmonic phonons in the self-consistent harmonic approximation: Theory and stochastic implementation. *Physical Review B* **96**, 014111 (2017).

[6] Bartók, A. P., Payne, M. C., Kondor, R. & Csányi, G. Gaussian approximation potentials: The accuracy of quantum mechanics, without the electrons. *Physical Review Letters* **104**, 136403 (2010).

[7] Giannozzi, P. *et al.* QUANTUM ESPRESSO: a modular and open-source software project for quantum simulations of materials. *Journal of Physics: Condensed Matter* **21**, 395502 (2009).

[8] Giannozzi, P. *et al.* Advanced capabilities for materials modelling with Quantum ESPRESSO. *Journal of Physics: Condensed Matter* **29**, 465901 (2017).

[9] Dal Corso, A. Pseudopotentials periodic table: From H to Pu. *Computational Materials Science* **95**, 337–350 (2014).

[10] Cowley, R. A. Anharmonic crystals. *Reports on Progress in Physics* **31**, 123 (1968).

[11] Baron, A. Q. R. Introduction to high-resolution inelastic x-ray scattering (2020). ArXiv:1504.01098.

[12] Gutierrez-Amigo, M. *et al.* Phonon collapse and anharmonic melting of the 3d charge-density wave in kagome metals. *Communications Materials* **5**, 234 (2024).

[13] Alkorta, M. *et al.* Symmetry-broken ground state and phonon mediated superconductivity in kagome CsV_3Sb_5 (2025). ArXiv:2505.19686, arXiv:2505.19686.

[14] Subires, D. *et al.* Order-disorder charge density wave instability in the kagome metal $(Cs,Rb)V_3Sb_5$. *Nature Communications* **14**, 1015 (2023).

[15] Li, H. *et al.* Observation of unconventional charge density wave without acoustic phonon anomaly in kagome superconductors AV_3Sb_5 (A = Rb, Cs). *Physical*

Review X **11**, 031050 (2021).
